# A Network Approach to Bipolar Symptomatology in Patients with Different Course Types

**DOI:** 10.1371/journal.pone.0141420

**Published:** 2015-10-27

**Authors:** M. A. Koenders, R. de Kleijn, E. J. Giltay, B. M. Elzinga, P. Spinhoven, A. T. Spijker

**Affiliations:** 1 Leiden University, Institute of Psychology, Section of Clinical Psychology, Leiden, The Netherlands; 2 PsyQ Rijnmond, Department of mood and anxiety disorders, Rotterdam, The Netherlands; 3 Leiden University Medical Center, Department of Psychiatry, Leiden, The Netherlands; 4 Leiden University, Institute of Psychology, Cognitive Psychology Unit, Leiden, The Netherlands; Maastricht University, NETHERLANDS

## Abstract

**Objective:**

The longitudinal mood course is highly variable among patients with bipolar disorder(BD). One of the strongest predictors of the future disease course is the past disease course, implying that the vulnerability for developing a specific pattern of symptoms is rather consistent over time. We therefore investigated whether BD patients with different longitudinal course types have symptom correlation networks with typical characteristics. To this end we used network analysis, a rather novel approach in the field of psychiatry.

**Method:**

Based on two-year monthly life charts, 125 patients with complete 2 year data were categorized into three groups: i.e., a minimally impaired (n = 47), a predominantly depressed (n = 42) and a cycling course (n = 36). Associations between symptoms were defined as the groupwise Spearman’s rank correlation coefficient between each pair of items of the Young Mania Rating Scale (YMRS) and the Quick Inventory of Depressive Symptomatology (QIDS). Weighted symptom networks and centrality measures were compared among the three groups.

**Results:**

The weighted networks significantly differed among the three groups, with manic and depressed symptoms being most strongly interconnected in the cycling group. The symptoms with top centrality that were most interconnected also differed among the course group; central symptoms in the stable group were elevated mood and increased speech, in the depressed group loss of self-esteem and psychomotor slowness, and in the cycling group concentration loss and suicidality.

**Conclusion:**

Symptom networks based on the timepoints with most severe symptoms of bipolar patients with different longitudinal course types are significantly different. The clinical interpretation of this finding and its implications are discussed.

## Introduction

Bipolar disorder (BD) is a chronic and highly disabling disorder that is characterized by constant risk of recurrence, despite receiving treatment according to contemporary practice guidelines [1, 2]. Course patterns seem to differ strongly between BD patients. From the few studies that specifically tried to identify different course types in patient groups receiving treatment, it appears that roughly three course types can be distinguished: 1) predominantly depressed, 2) episodic or cycling pattern, and 3) minimally impaired [3–6]. The current classification of BD into type I and II does not reflect these delicate distinctions in course patterns in sufficient detail [4, 7, 8]. The three course types have clinical face validity, and have been associated with specific clinical and prognostic characteristics. For instance, patients with cycling pattern were shown to have more life-time and family history of substance abuse, a worse long term course, and more severe disability [3,4,9]. A predominant depressive course was associated with more psychiatric comorbidity and a worse treatment response [10–12], whereas the relative stable BD patients were more often those who responded well to (pharmacological) treatment, who suffered less from comorbid disorders, and more often had a history of a ‘classic bipolar pattern’ of mania followed by depression [13,14].

However, the prediction of future course patterns remains challenging. Still, the strongest and most consistent predictor for future polarity and severity of the disease course is the previous polarity and severity [1, 9–11]. It is evident that symptomatology, cycling pattern, severity and polarity of episodes strongly differ between BD patients [3, 4], whereas recurrent episodes within a patient may show a highly similar pattern [1, 9, 10]. This implies that the vulnerability for developing a specific pattern of symptoms is rather consistent over time. In clinical practice the clinician, patient and his or her loved ones may recognize such warning symptoms already in an early phase, as the symptoms and their patterns are typical for that individual patient. However, it is difficult to get a systematic and statistical grasp of such observations using conventional epidemiological and statistical methods. In a recently developed network approach for psychopathology, emphasis is put on clusters and patterns of symptoms rather than on overall symptom severity. Some researchers have even suggested that psychiatric disorders can be defined as systems of causally connected symptoms [12, 13]. The basic assumption of this theory is that an underlying common (genetic or neurobiological) cause or generic latent variable has not been identified for psychiatric disorders such as BD [14]. Within the network approach, the collection of symptoms should be considered to be the disorder itself. This is opposed to most medical conditions in which symptoms are the expression of an underlying disease, such as a tumor or an inflammation in which the medical condition (e.g. the tumor) are directly responsible for its symptoms (e.g., headache, nausea etc.). Following this proposition, Borsboom and colleagues [13] suggested that studying symptom patterns and their complex intercorrelations will lead to more insight and understanding of psychiatric disorders.

Furthermore, using such an approach it is possible to identify those symptoms that are centrally positioned in the network and are more often and more strongly connected to other symptoms. An example of a simple network approach might be found in a BD patient with one night of shortened sleep duration, which results in increased energy and restlessness in patient A, but is strongly connected to loss of energy and feelings of sadness in patient B. Using a network approach within different bipolar course groups, such clinically important differences can be taken into account. The potential value of this approach has already been shown in previous studies. In a study by Cramer et al. [15] different networks were compared based on these network characteristics and it was shown that symptom networks behaved differently in subjects that experienced distinct stressful life events.

In a recent study by Goekoop et al. [16] the authors showed that within an unselected group of psychiatric patients, symptoms of a wide variety of disorders are closely interconnected, with particular groups of symptoms clustering together into 6 distinct psychiatric syndromes: depression, mania, anxiety, psychosis, retardation and behavioral organization. In line with these approaches, the purpose of the current study is to investigate whether BD patients with different longitudinal course patterns display differences in their symptoms networks. We hypothesize that specific symptoms play a central role in the network and therefore are more strongly connected to other symptoms in the network. These ‘central’ symptoms function as a ‘bridge’ between other more peripheral symptoms in the network and might differ across the three different course groups. Insight into these different symptom structures may lead to a deeper understanding of mood states and shifts in BD, and ultimately symptom patterns may serve as predictors of the future disease course.

## Method

### Ethical Statement

All patients participating in this study provided both written and verbal informed consent.

The study protocol was approved by the Medical Ethical Committee Mental Health Care Organisations Rotterdam (number: 7220) and the Central Committee Human Studies (number: NL18286.097.07) and was carried out in accordance with the Declaration of Helsinki.

### Participants

The data for this study consisted of a subset of patients from a 2-year prospective follow-up study among 173 bipolar outpatients, treated for BD by the Outpatient Clinic for Mood Disorders in The Hague (The Netherlands), with a diagnosis of BD I or BD II according to DSM-IV-TR diagnostic criteria.

All BD patients at the outpatient clinic were invited to participate in the study. After written informed consent was obtained, 173 patients were willing to participate in the follow-up study. Participants were older than 18 years and exclusion criteria in this study were schizo-affective disorder, neurological disease and substance abuse disorders.

Diagnoses of BD were based on DSM-IV criteria and were assessed with a standardized diagnostic interview [17] using the Dutch version of the MINI International Neuropsychiatric Interview Plus (version 5.00-R; MINI-PLUS), which has good interrater (kappa > .75) and test-retest reliability (kappa > .75) [17, 18]. The Questionnaire for Bipolar Illness, Dutch translation [19, 20] was used to specify subtypes of BD. After completing the baseline assessment, patients had face-to-face contacts with the research assistant at 3-, 6-, 9-, 12-, 15-, 18-, 21-, and 24- months follow-up.

Of the 173 patients participating in the follow-up study, 125 patients completed all 8 assessments and were selected for the current study. There were no significant differences in baseline demographic and clinical characteristics between the group who participated until the end of the study and the group that dropped out during the study and no differences in the longitudinal disease course were found.

### Procedure

#### Monitoring the longitudinal mood course

**Fig 1:**
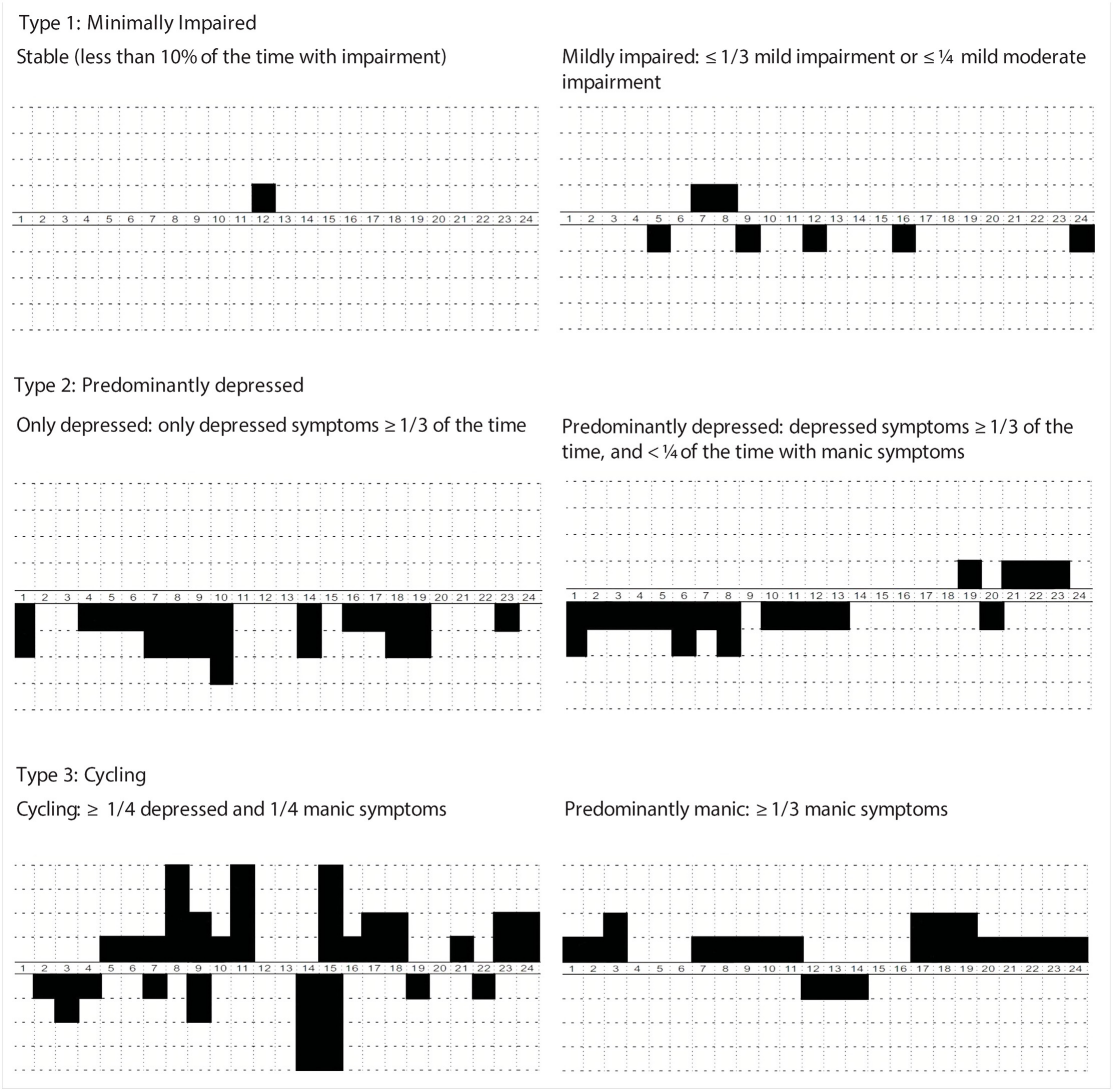
Example of course groups based on LCM data.

The NIMH monthly retrospective life chart method (LCM-r) [21, 22] was used at all 8 assessment sessions, to measure monthly functional impairment arising from manic or depressed symptoms during the previous 3 months. The LCM-r distinguishes four levels of severity for both mania and depression: (1) mild, (2) moderately low, (3) moderately high, and (4) severe. For every patient the 24 month life chart data were sorted for their overall course pattern based on proportion of time in a certain mood state and severity criteria. Proportion of time in depressive or manic mood state was calculated by counting the number of months with depressive or manic impairment divided by the total 24 months, which is a frequently used method [23]. In case of mixed mood states we followed the LCM Manual [24], which states that in case of mixed mood states both the rated mania and depression score are included in the calculation of specific course variables. Based on earlier studies [3, 4] we distinguished three different course groups. Fig 1 shows examples of life charts of patients in the different course groups. All patients fall within the scope of one of the three categories:

1. Mildly impaired: stable mood for more than 90% of the time, or reporting mild depressive/manic impairment ≤ 1/3 of the time or mild up to moderate depressive/manic impairment ≤ 1/4 of the time.
2. Predominantly depressed: depression related impairment on the LCM for more than 1/3 of the time with mild to mild moderate depressive symptoms, or depression related impairment at least 1/4 of the time with at least one month exceeding high moderate severity. Manic impairment should not be more than 1/4 of the time without exceeding mild severity.
3. Cycling: > 1/4 of the time manic impairment and > 1/4 of the time depression impairment with at least one month with mild moderate impairment (depression and mania) or with at least 1/8 of the time manic and 1/8 depressed impairment with at least one month with severity levels exceeding high moderate. Patients with predominantly manic or mixed symptoms (≥ 1/3 of the time with manic/mixed symptoms and < 1/4 of the time depressed symptoms) were also included in this group.

#### Manic and depressed symptoms

For the assessment of manic and depressed symptoms the Quick Inventory of Depressive Symptomatology- Self Report (QIDS-SR) [25], and the observer-based Young Mania Rating Scale (YMRS) [26] were administered. Both the QIDS and the YMRS have good (interrater) reliability and validity [25, 26]. Since the data used for the current study are part of a longitudinal study, both the QIDS and YMRS were assessed at 5 different timepoints (at baseline and subsequently every 6 months). At study entry, most patients were euthymic, so very low QIDS and YMRS scores were obtained at baseline, making this timepoint less suitable (because of small variance) for the current analyses. Since we aimed to investigate how symptoms are connected when these are actually present in BD patients, for every patient we selected the timepoint at which they were most symptomatic. Selecting the most symptomatic timepoint has largely to do with the fact that bipolar patients appear to be non-symptomatic for an ample proportion of the time: patients are 50% of the time symptom free (euthymic), 35% of the time in a depressed state and 10% of the time in manic state [27]. This means that when mood is measured at a random timepoint, a large proportion of the patients will report no or very slight symptoms only. This is also observed in the current study; on every timepoint a proportion of 35% to 40% of the patients appears to be euthymic. However, in order to ensure that networks are rather stable we will additionally perform sensitivity analyses over the average symptom scores on the 5 timepoints and report on this in the Results section. To this end, for all 3 course groups we will calculate edge weights between symptoms on all 5-timepoints separately and average these edge weights to construct 3 symptom networks that represent the average correlation network of symptom scores on all 5 timepoints. Subsequently we will compare these ‘full-data’ networks with the networks containing only the most severe symptom scores.

To determine the timepoint with most severe symptoms, YMRS and QIDS total scores were standardized and summed, and for every patient the timepoint with the highest total score was selected. For network analyses the raw item scores on the QIDS and YMRS of that specific timepoint were used.

In total the QIDS and YMRS consist of 27 items. Response scales on the QIDS and YMRS consist respectively of four and five categories. Some of the items assessed by the QIDS form separate domains (e.g. sleep) and several items of the QIDS and YMRS are overlapping. The items to which this applies were recoded if necessary and summed up in order to create symptom variables for the network analyses. In S1 Table it is shown what overlapping items were used to compose these new variables. The overlapping YMRS and QIDS item scores were standardized and the highest score was selected to represent the combined item. For the network analyses standardized scores were transformed back into raw scores.

Further, on three items (libido, lack of insight and appearance) of the YMRS a majority (≥ 94% of the participants) responded negatively (zero), leading to an unsuitable distribution for correlation analyses, therefore these items were not included in the analyses. The above mentioned adjustments resulted in a final total of 14 items that were included in the network analysis: irritability, increased speech, elevated mood, appetite/weight, restlessness, suicidality, concentration, self-esteem, interest, depressed mood, sleep duration, slowness, energy decrease, and sleep quality.

### Statistical Procedure

For comparison of baseline and clinical characteristics between the different course groups ANOVAs or Chi-square analyses were used. These analyses were performed using IBM SPSS for Windows (version 21.0; SPSS, Inc. Armonk, NY). Network analyses were performed using R 3.1.1 [28] with the igraph 0.7.1 package [29].

#### Network metrics

We constructed complete, weighted graphs (or networks) for each of the three course groups. In an unweighted graph, connections between two nodes are either present or absent. In weighted graphs, these connections are assigned a weight, representing the strength of the connection. Within the weighted graphs, the 14 symptom items function as nodes, and edge weights are defined as the groupwise Spearman’s rank correlation coefficient between the relevant items. The reason for using Spearman’s rank correlation coefficient instead of the polychoric correlation coefficient is the assumption of the normally distributed, continuous nature of the latent variables underlying the data in the latter case. We believe that this assumption is not met due to the wording of the questionnaire items on which the networks are based, as well as the non-normal distribution of the data.

#### Node metrics

There are several metrics that are informative about the properties of a network. It is important to mention that the output of network analyses is mainly discussed in a descriptive fashion, also in previous studies [15]. This means that the below mentioned metrics describe differences among the three course groups in terms of ‘stronger-weaker’ or ‘higher-lower’

The following three metrics are discussed in a descriptive fashion in the results section.

1. *Network density*. This reflects how interconnected a network is. For fully connected networks (like those in the current study) where the edge weights are based on a correlation matrix, this measure is equivalent to the arithmetic mean of the correlation coefficients. In other words, it is a measure of the connectivity of a network’s symptoms.
2. *Degree of centrality*. This reflects network characteristics on the node or symptom level. The *degree of centrality* is often referred to as node strength. With this measure, symptoms that have high degree centrality (e.g. symptoms that are most strongly connected to all other symptoms in the network) can be identified. Clinically this means that when a patient develops symptoms with high centrality, it will become more likely that other symptoms will emerge as well, since the central symptom is so strongly connected to other symptoms in the network [13].
3. *Random-walk betweenness*. An alternative way to look at the importance of a symptom is to look at route length or time it will take information (or symptomatology) to spread from a given node to others in the network, which is known as *betweenness centrality*. To this end, one could calculate the *shortest path* between two symptoms. However, as noted by Newman (2005), this assumes that information is purposefully directed towards the shortest route. In real-world situations, information wanders around more randomly. To account for this, Newman suggested an alternative measure of betweenness based on random walks. This *random-walk betweenness* of a node is defined as the number of times that a node is encountered on a random walk between two other nodes. This will give insight in how often a specific symptom lies on the path between two other symptoms and therefore might be seen as a bridge symptom that is likely to be ‘passed’ to reach other symptoms.

To be able to compare network density and centrality measures (degree centrality and betweenness) of the different networks we bootstrapped 95% confidence intervals (CI) using 10.000 iterations.

## Results

### Baseline Characteristics

Basic demographic and clinical characteristics of both the total sample and the separate course groups are summarized in Table 1. The mildly impaired, depressed and cycling group consisted of respectively 47 (37.6%), 42 (33.6%) and 36 (28.8%) patients. Mean age was 50.6 (SD = 11.2) years and patients were predominantly female (60.0%). Mean YMRS and QIDS scores used for network analyses were 3.1 (SD 5.3) and 10.2 (SD 5.0), respectively, indicating overall light mania scores and mild depressive symptoms at the most symptomatic timepoint.

**Table 1:** Baseline and clinical characteristics of the total sample and separate course groups.

	Total (N = 125)	Mildly impaired (N = 47)	Depressed (N = 42)	Cycling (N = 36)	P-value
Male sex; n(%)	50 (40.0)	20 (42.6)	17 (40.5)	13 (36.1)	.836
Mean age (SD)	50.6 (11.2)	53.1 (10.8)	51.9 (11.7)	45.8 (10.0)	.008
**Level of education, N (%)**					
- primary	25 (20.0)	8 (17.0)	9 (21.4)	8 (22.2)	.837
- secondary	40 (32.0)	18 (38.3)	15 (35.7)	7 (19.4)	.140
- higher	59 (47.2)	20 (42.6)	18 (42.9)	21 (58.3)	.308
**Clinical characteristics**					
BD I; N (%)	90 (72.0)	31 (66.6)	32 (73.2)	27 (75.0)	.502
Age of onset first (hypo-) mania; mean (SD)	30.5 (10.2)	32.5 (10.9)	29.9 (10.5)	28.8 (8.9)	.284
Age of onset first depression: mean (SD)	27.3 (9.9)	28.6 (10.6)	27.6 (10.3)	25.1 (8.0)	.323
**Medication use baseline; N (%)**					
Lithium	91 (72.8)	37 (78.8)	30 (71.4)	24 (66.7)	.459
Anti-epileptics	28 (22.4)	9 (19.1)	10 (23.8)	9 (25.0)	.789
Anti-psychotics	39 (31.2)	12 (25.5)	13 (31.0)	14 (38.9)	.462
Benzodiazepines	32 (25.6)	8 (17.0)	14 (33.3)	10 (27.8)	.199
Antidepressant	42 (33.6)	13 (27.7)	17 (40.5)	12 (33.3)	.442
**Mood/impairment during follow up**					
QIDS (highest score for network)	10.2 (5.0)	7.1 (3.6)	12.9 (4.2)	11.1 (5.4)	< .001
YMRS (highest score for network)	3.1 (5.3)	3.3 (5.4)	1.8 (3.4)	4.4 (6.7)	.108
Number of months depressive impairment LCM	7.6 (5.5)	2.7 (2.1)	12.2 (4.5)	7.7 (5.5)	< .001
Number of months manic impairment LCM	3.3 (4.7)	1.4 (1.9)	1.3 (2.0)	3.3 (4.5)	< .001

There were some significant differences among the 3 course groups. First, the cycling group was significantly younger than the two other groups (*F* = 5.1, *p* = .008). Furthermore, as expected (since the groups are classified based on the LCM mood data), the groups significantly differed on number of months in manic and depressed mood state. The depressed group had significantly more depressed months than the other two and the cycling group had significantly more manic months on the LCM.

Furthermore, the minimally impaired group displayed significantly lower mean QIDS score than the depression and cycling groups. We also compared (ANOVA) mean scores on all 14 symptom items (S1 Table). Symptoms that were more severely prevalent in the cycling and depressed group compared to the minimally impaired group were ‘depressed mood’, ‘self-esteem’, ‘loss of interest’, ‘lack of energy’ and ‘slowness’. On none of the symptoms significant differences in severity were found between the between the cycling and depressed groups.

### Network Density, Structure and Differences

**Fig 2:**
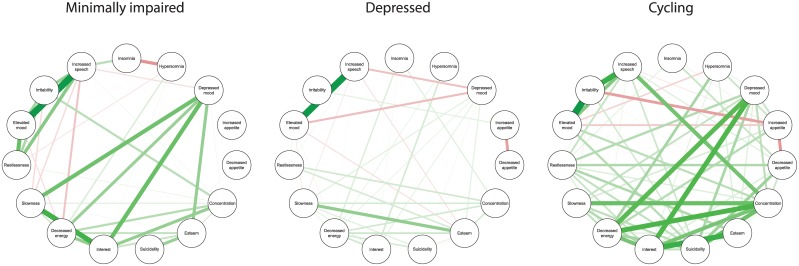
Weighted networks with manic and depressive mood symptoms for the three bipolar disease course groups. Each symptom reflects a node in the network. Connections between the nodes are Spearman's rank correlation coefficients (green: positive correlation coefficient, red: negative correlation coefficient) based on the timepoint with most severe symptoms. Correlation coefficients lower than ρ = .2 are not shown.

Fig 2 shows the pruned correlation networks (only moderate to strong connections (ρ > 0.2) between symptoms are shown) of the 3 course groups. The minimally impaired, depressed, and cycling groups had weighted network densities and 95% confidence intervals (CI) of respectively .186 (95% CI: .166–.185), .171 (95% CI: .131–.166), .250 (95% CI: .198–.269). These results implicate that symptoms were most strongly connected in the cycling group and least densely connected in the depressed group. Since the CIs of the three groups do not overlap it appears that the 3 groups differ significantly with respect to the overall connectivity of the symptoms.

### Degree Centrality of Bipolar Symptoms

**Fig 3:**
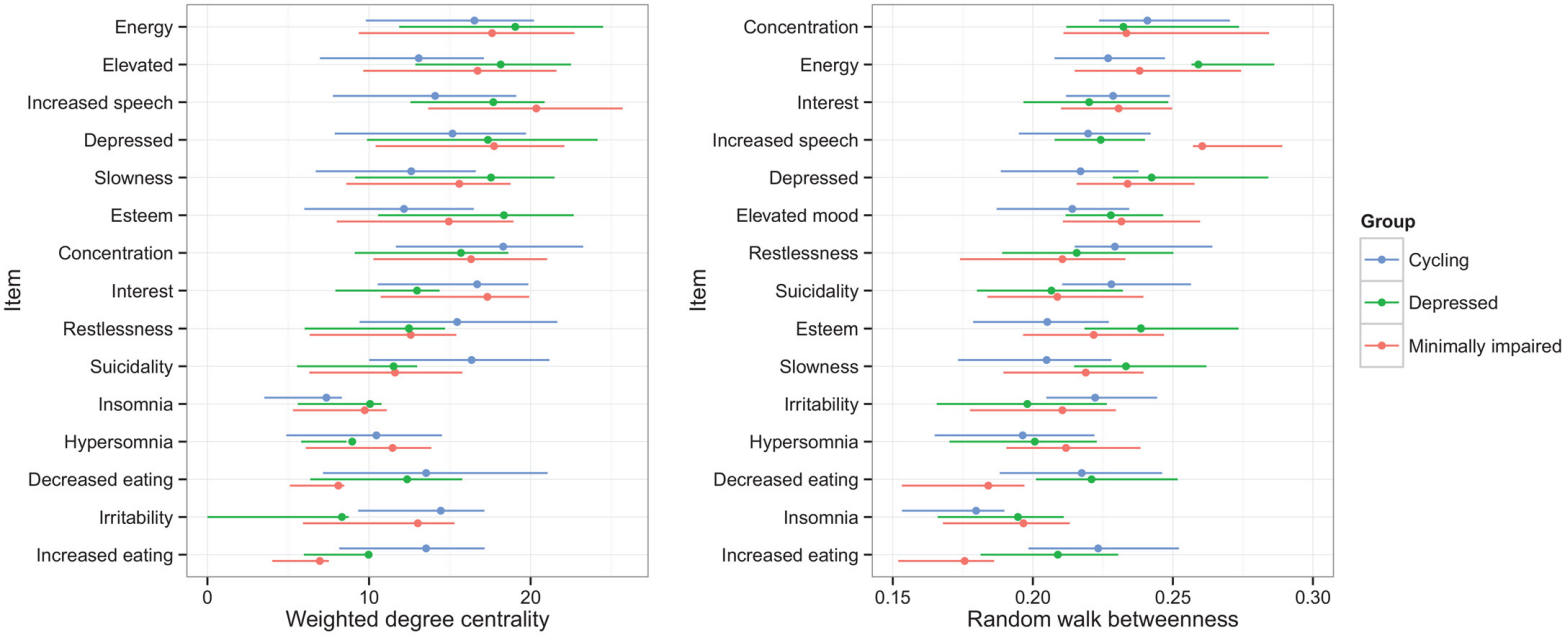
Differences in centrality and betweenness in the 3 course groups: minimally impaired compared to depressed and cycling group.

As mentioned before, how strongly specific symptoms are connected to other symptoms can be measured by their weighted degree centrality. In Fig 3 the weighted degree centrality and the CI of the symptoms in the different networks is depicted. Fig 3 shows the differences in centrality between the 3 groups. Table 2 shows the 5 items with the highest centrality within every group.

**Table 2:** Five items with highest weighted degree centrality in the different course groups.

Mildly impaired	Depressed	Cycling
*Item*	*Item*	*Item*
Increased speech	Loss of energy	Concentration
Loss of interest	Elevated mood	Loss of interest
Depressed mood	Decr. self-esteem	Loss of energy
Loss of energy	Increased speech	Suicidality
Elevated mood	Slowness	Restlessness

First, based on centrality, the minimally impaired and depressed groups seemed to have most in common, and centrality strength of several symptoms seemed to overlap. When overlap of CI errorbars is about less than 50% (based on visual inspection) these can be considered significantly different [30]. This means that within the cycling group especially the symptoms ‘restlessness’ and ‘suicidality’ had high centrality compared to the other two groups. In the minimally impaired group ‘increased speech’ and ‘loss of interest’ were the most central symptoms compared to the other groups, although overlap for these two symptoms was more than 50% with at least one of the other groups. Symptoms ‘decreased self-esteem’ and ‘slowness’ were distinct central symptoms for the depressed group. In all three groups ‘loss of energy’ was a highly central symptom.

### Random Walk Betweenness

In Fig 3 the random-walk betweenness and the CI’s of the symptoms in the different networks is depicted, and Table 3 shows the 5 items with the highest random-walk betweenness per group. The symptoms with high random-walk betweenness were rather comparable to the symptoms with high degree centrality, especially in the depressed and cycling group.

**Table 3:** Five items with highest random walk-betweenness in the different course groups.

Mildly impaired	Depressed	Cycling
*Item*	*Item*	*Item*
Increased speech	Loss of energy	Concentration
Concentration	Decr. self-esteem	Loss of energy
Elevated mood	Concentration	Suicidality
Loss of interest	Slowness	Depressed mood
Loss of energy	Loss of interest	Loss of interest

### Sensitivity Analyses

For reasons described in the method section we chose to select the timepoint with most severe symptoms to compose our networks from. However, it is important to test whether the networks are not biased because of this specific selection of the timepoint. Therefore we performed sensitivity analyses based on all 5 timepoints. For all three groups symtpoms correlations were calculated on every timepoint and the average edge weights were were used to construct the new networks (S1 Fig). The minimally impaired, depressed, and cycling groups had weighted network densities and 95% confidence intervals (CI) of respectively .159 (95% CI: .114–.191), .141 (95% CI: .113–.164), .227 (95% CI: .185–.263). These results show that network densities of the networks based on all timepoints are comparable to the ‘severe score networks’, since again the cycling network is most dense and symptoms in the depressed network are least densely connected. Further, the network figures indicate that the average symptom scores lead to rather comparable network structures, although some additional associations appear.

## Discussion

In the current study we approached the challenge of identifying mechanisms for the development of different bipolar course types from a novel methodological angle. With the use of a new approach in psychiatry, differences in symptom networks were studied within a large longitudinal cohort of BD patients with different course types. Our main finding was that symptom networks significantly differed between BD patients that were either minimally impaired, predominantly depressed or cycling over a two-year period. Descriptive comparison of the networks also indicated that symptoms that play a central role in the network differ across the groups. These findings and their clinical interpretation will be discussed in more detail in the following sections.

### Differences in Symptom Networks between the Course Groups

The symptoms networks of the three groups differed most on overall connectivity of the network. Manic and depressed symptoms in the cycling group were most strongly interconnected compared to the depressed and minimally impaired group. This might imply that symptom patterns of patients in the cycling group are rather homogenous, meaning that most patients that display one symptom are very likely to suffer from the other densely connected symptoms at the same time.

The strong interconnection between manic and depressed symptoms might also explain the fact that the cycling patients are more prone to display mixed mood states and switch from one mood state to the opposite mood state. The depressed group on the other hand, shows the least connected network, even though they do suffer from rather severe symptoms (especially compared to the stable group). The weaker connection between the symptoms might indicate that there is more heterogeneity in the symptoms patterns displayed by the depressed group. Unlike the other two groups, the depressed patients may have more diverse combinations of symptoms that can occur simultaneously probably due to individual differences. Although the sensitivity networks including all data-points over 2 years show the same density patterns for the three groups, the cross-sectional character of the data-points makes it difficult to determine whether the weaker connection between symptoms might also indicate that symptom states are less dynamic in the depressed group. Clinically, the ‘depression-prone’ bipolar patients indeed seem to be a less dynamic group in terms that they often display long-lasting or even chronic depressed states with less alternation between manic or euthymic states [3, 8, 27]. Time-lagged measurements are needed to confirm this hypothesis.

In addition, in the three course groups symptoms also differed in terms of centrality (degree centrality and random walk betweenness). These central symptoms are concentration loss and suicidality for the cycling group, loss of self-esteem and psychomotor slowness for the depressed group, and increased speech and elevated mood for the minimally impaired group. This means that these symptoms are most strongly connected to the other symptoms in the network. When these symptoms emerge it is highly probable that other symptoms occur as well. In the current sample central symptoms were not more prevalent in the specific course groups. However, in previous studies some of these symptoms have been more frequently associated with specific course patterns.

For instance, both central symptoms in the cycling group have been associated with adverse course patterns before. Several studies found higher prevalence of suicidality among patients with more previous episodes, rapid cycling patterns [31] and mixed episodes [32]. Patients that have more chronic and recurrent symptom patterns also show more cognitive impairment (including attentional/concentration problems) [33, 34].

In the depressed group, loss of self-esteem played a central role in the network. Self-esteem has been previously described as an important predictor of change in bipolar depression, suggesting that decreases in self-esteem are tightly linked to increases in other depressive symptoms [35], as was also displayed in the current analyses.

Finally, in the minimally impaired group the typically manic symptoms of increased speech and elevated mood are most consistently identified as central symptoms in the network. This finding is more difficult to interpret in light of previous literature. In this group it is likely that specific symptom patterns are more difficult to detect, since these patients simply display less severe manic and depressed symptomatology. However, one possible explanation for these specific symptoms patterns, might be that if these minimally impaired patients develop symptoms, this will more likely lead to the activation of the central hypomanic symptoms which are by definition associated with less functional impairment. However, since the minimally impaired group reported significantly less severe symptoms on many items, any differences with the other two groups should be interpreted with caution and may be due to differences in prevalence of manic and depressed symptoms.

### Similarities between the Course Groups

Although there are several important differences in symptoms centrality across the groups, decrease in energy level seems to be a highly central symptom in all three course groups. Thus, changes in energy levels are strongly associated with changes in other symptoms in all networks. In the light of the recent changes in diagnostic criteria of BD in the DSM-5 this is an interesting finding. The diagnostic A criterion for a (hypo-) manic episode (elation/euphoric or irritable mood in the DSM-IV) is now extended with the criterion that ‘the mood change must be accompanied by persistently increased activity or energy levels’. Because of the lack of empirical evidence, the validity of this additional criterion has been disputed [36, 37]. The current findings do show that changes in energy levels might be highly central symptoms in BD, but no direct evidence for a central role of energy increase is found here, since the currently found central energy decrease only reflects the energy decrease as a symptom of depression, and not mania. The symptoms ‘restlessness’ and ‘elevated mood’ reflect increases in (motor) energy but these symptoms are not identified as a central symptom in any of the networks.

Another important similarity between the networks is the fact that, across networks, symptoms do not form isolated ‘manic’ and ‘depressed’ symptom clusters, but symptoms of both poles are interconnected. This is in line with previous findings, showing that bipolar depression and mania do not occur as categorical as they are presented in diagnostic handbooks, but a substantial number of patients seems to experience episodes with manic and depressive admixtures (up to 65%) [38–40]. This more dimensional representation is now acknowledged in the DSM-5 with the option to apply a ‘with mixed features’ specifier to depressed or manic mood states. Moreover, these findings are also in line with previous studies using the network approach which challenged the current categorical approach of psychopathology by showing the interconnection between symptoms of different psychiatric disorders [16, 41]. The fact that manic and depressed symptoms are interconnected, overlap and often occur simultaneously implicates that mania and depression are not at all total opposite and distinct mood states, but lie on the same spectrum and presumably overlap with regards to their structure and connectivity strengths of the symptom networks. From such a network perspective one could hypothesize that stronger interconnection and overlap between manic and depressed symptoms (especially in the cycling group) explains a cycling pattern rather than a stable state of either deep depression or mania. The latter would have been expected in case of non-overlapping attractors. In BD decreased sleep, psychomotor agitation and irritability are symptoms associated both with mania and depression [40] and therefore possibly connecting both ends of the mood spectrum. For instance, decreased sleep could lead to increased energy levels and (hypo-) manic mood states, however after some time energy levels might eventually drop leading to a more depressed state. A transition from mania to depression might for instance be due to insomnia within a manic mood state due to feelings of grandeur. This overlapping insomnia symptom may activate depressive symptoms such as loss of energy, restlessness, and concentration problems, subsequently leading to a depressed state. However this interpretation is rather tentative and underlines the need for confirmation in other BD populations as well as longitudinal monitoring of symptoms and their temporal development.

### Strengths and Limitations

This is the first attempt to explain differences in bipolar mood course by investigating symptom associations through a network approach. This could be a first step in explaining the variety of course patterns that are clinically displayed by BD patients by investigating how bipolar symptoms interact and reinforce each other. Further, the current study is also novel to the extent that manic and depressed symptoms are not treated as separate constructs, but fitted into the same model.

However, some important limitations should be taken into account. First, patients were divided into three groups based on their longitudinal course patterns. Although these three specific course groups are previously described in the literature and roughly reflect what is observed in clinical practice, one could argue for the existence of many more different course groups [4]. Second, course data were assessed through monthly ratings on the LCM which provides a global impression of the disease course, but lacks detailed information about more subtle mood changes and every minor episode. Third, in the current outpatient sample severe mood states (especially manic symptoms) were rare, which also led to the exclusion of some symptom items because of low variance. This implicates that the current findings can only be translated to BD patients with relatively mild symptom severity. Fourth, we used the timepoint with most severe symptomatology to construct the symptom networks, which requires careful interpretation to prevent the trap of circular argumentation. As the minimally impaired group by definition had low symptom severity this may partly explain some of the differences, although the other two groups showed important distinctions in their networks, but not in symptom severity. Fifth, the use of Spearman’s rank correlation shows the global structure of the interconnections of symptoms, however different correlation techniques such as polychoric correlations, partial-correlation analyses or the Lasso procedure are valuable to use in future studies since they allow for a more refined exploration of the network structure [42]. Last, although a clinical sample of 125 patients is relatively large, for the analyses of 14 different variables within three groups of (on average) 40 patients, sample sizes are small. Due to low statistical power, the current results should be interpreted with caution and replication in larger samples is needed.

### Conclusion and Future Directions

The current approach might be a first step in investigating the mechanisms behind the development of different bipolar course patterns by looking at bipolar symptoms themselves. The current network structures are based on Spearman’s rank correlations and only show the global structure of the interconnections of symptoms, however different correlation techniques such as partial-correlation analyses or the Lasso procedure are valuable to use in future studies since they allow for a more refined exploration of the network structure [42]. Futher, longitudinal studies are needed to show whether symptom networks have truly predictive and clinical value. Within these studies follow-up measurement should be very frequent to allow for the detection of (directed) relations between bipolar symptoms. In the current study follow-up measurements for the QIDS and YMRS were 6 months apart, making studying of the development of symptoms over time impossible, hence the cross-sectional symptom networks. Novel approaches such as the Experience Sampling Method [43, 44] might be highly suitable for the measurement of moment-to-moment development of symptoms and by that gain insight in the causal chain in which symptoms interact in BD. A recent publication [45] already showed the value of ESM data in detecting temporal mechanisms in depressive symptom patterns and identifying so called ‘tipping points’ that indicate a downfall into a full mood episode. Identifying these patterns in bipolar patients might have great clinical value in predicting future mood course and ultimately the prevention of new mood episodes.

## Supporting Information

S1 Data SPSS Data file with data used for network analyses. (SAV)

S1 Fig Weighted networks with manic and depressive mood symptoms for the three bipolar disease course groups. Each symptom reflects a node in the network. Connections between the nodes are Spearman's rank correlation coefficients (green: positive correlation coefficient, red: negative correlation coefficient) based on the average edge weights between symtpoms of all 5 separate timepoints. Correlation coefficients lower than ρ = .2 are not shown.
(EPS)

S1 Table Mean scores on symptom items and differences between the 3 course groups. (DOCX)
