# Supplementary material for: A Network Approach to Bipolar Symptomatology in Patients with Different Course Types
Source: PLoS One. 2015 Oct 27;10(10):e0141420. doi: 10.1371/journal.pone.0141420 (PMC4624774; doi:10.1371/journal.pone.0141420)
Supplement: S1 Table — (DOCX) [file pone.0141420.s003.docx]

| **Course Groups** | **Minimally impaired (1)**  **Mean (SD)** | **Depressed**  **(2)**  **Mean (SD)** | **Cycling**  **(3)**  **Mean (SD)** | **P-value** | **Post hoc (Bonferroni)** |
| --- | --- | --- | --- | --- | --- |
| **Mood Symptoms** |  |  |  |  |  |
| **Elevated mood** (Y) | .36 (.82) | .24 (.58) | .44 (.77) | .455 | - |
| **Speech** (Y) | .60 (1.4) | .44 (1.10) | .83 (1.68) | .433 | - |
| **Depressed** (Q) | .40 (.54) | 1.51 (.78) | 1.22 (1.05) | <.001 | 1<2, 1<3 |
| **Esteem** (Q) | .43 (.80) | 1.63 (1.2) | 1.11 (1.04) | <.001 | 1<2, 1<3 |
| **Suicidality** (Q) | .32 (.63) | .68 (.85) | .53 (.74) | .086 | - |
| **Loss of interest** (Q) | .43 (.58) | 1.20 (.87) | 1.00 (.89) | <.001 | 1<2, 1<3 |
| **Loss of energy** (Q) | .81 (.92) | 1.49 (.68) | 1.33 (8.6) | <.001 | 1<2, 1<3 |
| **Slowness** (Q) | .37 (.74) | 1.10 (.97) | .86 (.93) | <.001 | 1<2, 1<3 |
| **Hypersomnia (Q)** | .72 (.90) | .61 (.74) | .89 (.95) | .329 | - |
| **Insomnia** | 2.09 (.90) | 2.26 (.60) | 1.86 (.93) | .143 | - |
| *Waking up too early (Q)* | .60 (.99) | .98 (1.11) | .44 (.65) |  |  |
| *Falling asleep (Q)* | .74 (1.10) | .95 (1.18) | 1.06 (1.12) |  |  |
| *Sleep decrease (Y)* | .36 (.92) | .17 (.54) | .33 (.83) |  |  |
| **Restlessness** |  |  |  | .076 | - |
| *Feeling restless (Q)* | .36 (.74) | .76 (.89) | 1.03 (1.00) |  |  |
| *Increased motor activity (Y)* | .38 (.82) | .22 (48) | .36 (.72) |  |  |
| **Concentration** | .98 (.79) | 1.40 (.79) | 1.28 (.94) | .053 | - |
| *Concentration (Q)* | .79 (.69) | 1.27 (.78) | 1.11 (.89) |  |  |
| *Thought disorder (Y)* |  |  |  |  |  |
| **Decreased weight/appetite** | .79 (1.02) | .79 (.95) | .81 (.92) | .995 | - |
| *Decreased appetite(Q)* | .19 (.45) | .29 (.51) | .47 (.61) |  |  |
| *Decreased weight (Q)* | .70 (1.02) | .63 (.94) | .61 (.93) |  |  |
| **Increased weight/appetite** | .40 (.89) | .64 (.93) | .67 (1.0) | .360 | - |
| *Increased appetite (Q)* | .15 (.51) | .32 (.52) | .47 (.94) |  |  |
| *Increased weight (Q)* | .30 (.78) | .46 (.93) | .39 (.73) |  |  |
| **Aggression/irritability** | .72 (1.2) | .29 (.84) | .89 (1.3) | .051 | - |
| *Irritability (Y)* | .60 (1.4) | .29 (.84) | .83 (1.3) |  |  |
| *Aggressive behavior (Y)* | .21 (.62) | .05 (.31) | .22 (.64) |  |  |
| *Not included* |  |  |  |  |  |
| *Libido (Y)* | .11 (.43) | .02 (.16) | .11 (.32) |  |  |
| *Lack of inside (Y)* | .06 (.32) | .00 (.00) | .06 (.33) |  |  |
| *Appearances (Y)* | .04 (.20) | .00 (.00) | .11 (.32) |  |  |
|  |  |  |  |  |  |

**Table S1** Mean scores on symptom items and differences between the 3 course groups

*Bold symptoms represent variables included in the network analyses. The variables ‘sleep duration’, motor activity’, ‘concentration’, ‘weight/appetite’, ‘aggression/irritability’ are composed of the italicized symptoms depicted below these variables. Whether symptoms reflect items from the YMRS (Y) or QIDS (Q) is displayed between brackets.*
